# Book Review: Music, Passion, and Cognitive Function

**DOI:** 10.3389/fnins.2017.00361

**Published:** 2017-06-23

**Authors:** Stephan T. Vitas

**Affiliations:** Behavioural Research ConsultantSilver Spring, MD, United States

**Keywords:** music, cognition, emotion, nervous system, culture, language, philosophy, experimental design

In this his latest opus, Professor Leonid Perlovsky offers a compendium of philosophical and mechanistic viewpoints on emotion and music, an anthropological-historical overview of the influence of music on development of culture, and implications for current experimental techniques of neurological investigation.

Emphasizing the cognitive effects of music, the author considers the evolution of consciousness, musical styles, and cultural development, detailing the split created by language in our inner and outer world, creating an existential grief which music comforts. Differentiating the concrete-semantic functions of language against the emotional-semantically vague atmosphere of music, the cognitive mechanisms developed by music overcome this dissonance and aid in the evolution of language, connecting us to ancient instincts, creating new emotions to bring about a synthesis, a wholeness in the psyche where new knowledge contradicts old knowledge.

Beginning with the ancient Greeks' desire to mediate emotions with reason, Perlovsky reviews the development of musical theory from that time in its attempts to harmonize disparate tendencies of emotion and thought into a coherent approach to living. Music influenced emotions for enjoyment via tonality through the centuries to the point where Helmholtz could add scientific analysis, enabling us to confirm music's inborn mechanisms by experimentation. The writer then discusses modern music theories, which do not answer the cognitive connection to his satisfaction, while regarding concepts as mechanisms of mental models from our perceptions. He follows the interplay of concepts with instincts, emotions, and subsequent behavior as we modify our knowledge base, contrasting and revising our evolving concepts with the outside world. Emotions express harmony or dissonance between knowledge and the world. Knowledge being a higher order operation, these emotions become aesthetic in nature, aiding in the advance of our sense of purpose (a survival function).

Cognition blends language and experience to summarize the world to our minds, with language distinguishing details of reality with experience. These details may, and often do, contradict, each with the other, discouraging a harmonic balance to unify meaning and purpose. The conflict between differentiation and synthesis is to be balanced by music if culture is to evolve further. Each new bit of knowledge creates a basic discomfort. Music, in the author's view, restores the unity of Self.

Dr. Perlovsky details experiments confirming the unification function of music and its benefits in assisting with knowledge acquisition in modern endeavors. To aid in experimental exploration he takes up the call for a Physics of the mind, aided by considerable advances in brain imaging equipment (MEG, MRI, PET, etc.). This should assist in discerning how the emotions of beauty relate to the meaning and purpose in our individual lives, and he details his experiment in aesthetic chills, quantifying a heretofore abstract concept to explain the effects of narratives and mythic structure.

He traces development of music and human consciousness from the ancients of the eastern Mediterranean through the familiar stages of Continental European music styles, delineating parallels with the changing focus of intellectual and personal perspectives on cognition and self-concept, mind and emotion, the social and the political, the ideational and the spiritual. Music is described as the harmonizing mechanism. He outlines differing arguments over concepts, emotions, personality types, and their varied processing of musical input. Contrasting operations of emotion in the personality vs. emotion in music are considered with their functions in cognition and language and ramifications for experimental design.

As we have seen the utility of music in rehabilitation for Alzheimer's, dementia, and traumatic brain injury, the elaboration of hypotheses into methods is vital to progress in cortical research, and as the author contends, for the further development of culture. This volume presents Dr. Perlovsky's arguments specific to his focus of experimental endeavor and provides a quantum step in deriving techniques for neurological aesthetic research and cultural theory.

Conveniently, an abstract provides a basic outline for each chapter, and bibliographies are provided specific to the chapters. Written in high scholarly style for students of behavioral aesthetics and researchers in neurophysiology and auditory cognitive neuroscience, this work affords a multi-disciplinary scope with a considerable quantity of theoretical subtlety. Presenting much to inspire further discussion and debate, it is meticulous in philosophical detail, rationales, as well as theoretical and procedural implications, a benchmark in the evolution of an idea and a method.

## Author contributions

The author confirms being the sole contributor of this work and approved it for publication.

### Conflict of interest statement

The author declares that the research was conducted in the absence of any commercial or financial relationships that could be construed as a potential conflict of interest.

